# Psychometric properties of the Patient Health Questionnaire-4 among Hong Kong young adults in 2021: Associations with meaning in life and suicidal ideation

**DOI:** 10.3389/fpsyt.2023.1138755

**Published:** 2023-03-09

**Authors:** Ted C. T. Fong, Rainbow T. H. Ho, Paul S. F. Yip

**Affiliations:** ^1^Centre on Behavioral Health, Faculty of Social Sciences, The University of Hong Kong, Pokfulam, Hong Kong SAR, China; ^2^Department of Social Work and Social Administration, Faculty of Social Sciences, The University of Hong Kong, Pokfulam, Hong Kong SAR, China; ^3^Centre for Suicide Research and Prevention, Faculty of Social Sciences, The University of Hong Kong, Pokfulam, Hong Kong SAR, China

**Keywords:** measurement invariance, mediation, psychological distress, meaning in life, Patient Health Questionnaire-4 (PHQ-4), suicidal ideation, young adults, scale validation

## Abstract

**Background:**

Young adults in Hong Kong are subject to elevated psychological distress given the societal stressors such as civil unrest and COVID-19 pandemic and suicide is a leading cause of death among them. The present study aimed to evaluate the psychometric properties and measurement invariance of the 4-item Patient Health Questionnaire-4 (PHQ-4) as a brief measure of psychological distress and its associations with meaning in life and suicidal ideation (SI) in young adults.

**Materials and methods:**

A mobile survey recruited a large and random sample of 1,472 young adults (Mean age = 26.3 years, 51.8% males) in Hong Kong in 2021. The participants completed the PHQ-4 and Meaning in Life Questionnaire–short form (MLQ-SF) for presence of meaning in life (MIL), SI, COVID-19 impact, and exposure to suicide. Confirmatory factor analysis was conducted to examine the factorial validity, reliability, and measurement invariance of PHQ-4 and MLQ-SF across gender, age, and distress subgroups. Multigroup structural equation model evaluated and compared the direct and indirect effects of latent MIL factor on SI *via* latent PHQ-4 factor across distress groups.

**Results:**

Both MIL and PHQ-4 supported a 1-factor model with good composite reliability (Ω = 0.80–0.86) and strong factor loadings (λ = 0.65–0.88). Both factors showed scalar invariance across gender, age, and distress groups. MIL showed significant and negative indirect effects (*αβ* = −0.196, 95% CI = −0.254 to −0.144) on SI *via* PHQ-4. PHQ-4 showed a stronger mediating role between MIL and SI in the distress group (Δ = −0.146, 95% CI = −0.252 to −0.049) than the non-distress group. Higher MIL predicted higher likelihoods of help-seeking (Odds ratios = 1.46, 95% CI = 1.14–1.88).

**Conclusion:**

The present results support adequate psychometric properties in terms of factorial validity, reliability, convergent validity, and measurement invariance for the PHQ-4 in young adults in Hong Kong. The PHQ-4 demonstrated a substantial mediating role in the relationship between meaning in life and SI in the distress group. These findings support clinical relevance for using the PHQ-4 as a brief and valid measure of psychological distress in the Chinese context.

## Introduction

Hong Kong has undergone both civil unrest and COVID-19 pandemic in the past years and these events act as societal stressors and predispose citizens to elevated levels of mental distress (1). Suicidal behavior (SB) is a leading cause of death among youths and young adults in Hong Kong (2). SB in adolescents and young adults is a global challenge that carries clinical relevance to various socio-economic factors (3) and reduced life expectancy (4). Epidemiological surveys in Hong Kong (5, 6) found the prevalence of probable depression and suicidal ideation (SI) to range from 11.2 to 25.7% and 4.3 to 9.1% among respondents sampled between 2019 and 2020. Common risk factors of SB in youths included depression, mental disorders, psychosocial stressors, and exposure to suicide (7–9).

The Interpersonal Theory of Suicide (10, 11) states that experiences of perceived burdensomeness and thwarted belongingness contribute to SI which could lead to SB and suicide attempt (SA) (12). Presence of meaning in life (MIL) refers to the subjective sense that one’s life is meaningful and Kleiman and Beaver (13) proposed MIL as a potential resiliency factor of SI. Recent studies have associated presence of MIL with lower psychological distress (14, 15) and lower risks of SI among adolescents (16, 17). Higher presence of MIL was associated with lower levels of SI and SB among suicidal patients attending a psychiatric emergency department (18). Costanza, Amerio (19) have postulated that MIL could mediate the relationships between perceived burdensomeness and thwarted belongingness with SI.

The Patient Health Questionnaire (PHQ-9) is a 9-item self-report measurement tool of depressive and somatic symptoms with good construct validity and diagnostic ability of major depression (20–22). The shortened 4-item version of the PHQ-9 (PHQ-4) is an ultra-brief screening tool of mental distress over the past 2 weeks (23). The PHQ-4 comprises two items each from the PHQ-9 and the Generalized Anxiety Disorder scale. Though the PHQ-4 has been validated in cultural contexts such as Greek, Korean, and German samples (24–26), no existing studies have validated the psychometric properties of the PHQ-4 in the Chinese context. Besides, it is important to examine the stability of the measurement parameters of PHQ-4 across demographic groups. Equality of factor loadings and item thresholds are prerequisites to compare the latent mean of PHQ-4 factor across groups in an unbiased manner (27). Despite the established relationships among MIL, psychological distress, and SI, no existing studies have examined the potential mediating role of PHQ-4 between MIL and SI.

In light of the research gaps, the present study conducted a systematic evaluation of the psychometric properties of the PHQ-4 in a large sample of Chinese young adults in Hong Kong. This study had several hypotheses. In Hypothesis 1, the PHQ-4 and MIL factors would show satisfactory factorial validity and reliability. In Hypothesis 2, both factors would show measurement invariance across demographic groups. In Hypothesis 3, the PHQ-4 factor would display adequate convergent validity in terms of negative correlations with MIL and positive correlations with COVID-19 impact, exposure to suicide, and SI. In Hypothesis 4, the PHQ-4 would partially mediate the relationship between MIL and SI. The first objective was to examine the factorial validity and reliability of the PHQ-4 and MIL. The second objective was to test the measurement invariance of the PHQ-4 and MIL across gender, age, and distress groups. The third objective aimed to examine the mediating role of PHQ-4 in the relationship between MIL and SI. The fourth objective was to examine the associated factors of help-seeking behaviors among distressed respondents.

## Materials and methods

### Study design and procedures

The present cross-sectional study recruited young adults under random sampling *via* a phone survey. The survey was part of the surveillance research on the youth mental wellness under the OpenUp project. OpenUp is a 24-h emotional support program that provides online text-based counseling service for youth and young adults (28, 29). Inclusion criteria included residence in Hong Kong, aged between 18 and 36, able to understand spoken Cantonese. The telephone survey was administered by the Social Science Research Centre in the University of Hong Kong. Using mobile number prefixes published by the Office of the Communications Authority in Hong Kong, a random sample of 125,746 mobile phone numbers was generated. The computer assisted telephone interviewing system was used to contact potential respondents from 18:30 to 22:30 in the weekdays. The telephone interviewers were fluent in Cantonese and received prior trainings for conducting the interviews. Robust quality control was used to check the validity of telephone interviews for self-report measurements *via* audio recording and random callbacks.

The survey successfully completed 1,501 phone interviews between August and December 2021. The survey response rate was 68.9% with 544 refusals and 132 uncompleted interviews. The final study sample was 1,472 respondents after excluding 29 participants who were aged below 18 years. The participants provided oral informed consent before answering a self-report questionnaire on phone in around 5 min. Participation in the survey was voluntary and all information provided by the participants was kept strictly confidential. The respondents were provided contact information of 24-h hotlines and online services for emotional support in case they felt distressed during the interview. Ethical approval for this project was obtained from the Human Research Ethics Committee of the author’s university (Reference number: EA1709039).

### Measures

The PHQ-4 is an international brief screening scale for anxiety and depressive symptoms (23) and has been used widely in the general population (24, 26, 30) and various clinical samples (25, 31–33). This scale assessed four symptoms of mental distress of the respondents over the past 2 weeks. An example item was “feeling down, depressed or hopeless.” The items were answered on a 4-point Likert scale from 0 = “not at all” to 3 = “nearly every day.” The Chinese version of the PHQ-9 has been validated in a community sample of 1,045 adults in China (34). The total PHQ-4 score has a theoretical range from 0 to 12, with higher scores denoting greater distress. In the present sample, all four PHQ-4 items showed positive skewness (0.81–1.58) and substantial floor effects with 30.4–62.4% of the respondents endorsing the minimum category. They were treated as ordinal categorical variables in subsequent analyses.

Meaning in life was assessed by the Presence subscale of the 6-item short form of the Meaning in Life Questionnaire (MLQ-SF) (35). This subscale consisted of three items on the presence of MIL. An example item was “My life has a clear sense of purpose.” The three items were rated on a 7-point Likert scale from 1 = “absolutely untrue” to 7 = “absolutely true” and the total MIL score were derived by averaging the three item scores. In the present sample, the three MLQ-SF items followed a normal continuous distribution (skewness = −0.30 to 0.05) without any floor or ceiling effects. The Chinese version of the MLQ-SF has recently been validated in student samples in Hong Kong (36).

The questionnaire inquired demographic characteristics such as gender, age, and student status. The impact of COVID-19 was measured by asking the respondents the extent they were distressed by the pandemic on a 5-point Likert scale from 1 (not at all) to 5 (severe). SI was assessed by asking whether they had considered suicide in the past 12 weeks; and exposure to suicide was assessed by asking whether they personally knew someone who attempted suicide before. The respondents were asked whether they encountered any distressing issues in the past 4 weeks, and if yes, they were asked a follow-up question on whether they had sought help for the distressing issues (37).

### Data analysis

Descriptive statistics and polychoric correlations of the MLQ-SF and PHQ-4 items were obtained. The dataset had minimal missing data except for one MIL and PHQ-4 item. Missing data were handled using full information maximum likelihood under the missing-at-random assumption (38). Psychometric properties of the PHQ-4 and MLQ-SF were examined in four steps. First, their factorial validity was evaluated by confirmatory factor analysis (CFA). The 2-factor CFA model was estimated under the robust weighted least square estimator in Mplus 8.6. One residual covariance was added between the two anxiety items of the PHQ-4 based on theoretical justifications. Model fit was evaluated using the following criteria on the fit indices (39): comparative fit index (CFI) ≥ 0.95, Tucker-Lewis index (TLI) ≥ 0.95, root-mean-square error of approximation (RMSEA) ≤ 0.06, and standardized root mean square residuals (SRMR) ≤ 0.06. Composite reliability of the factors was evaluated *via* McDonald Omega (Ω) with values of at least 0.75 indicating good reliability.

The second step evaluated the measurement invariance of the PHQ-4 and MLQ-SF across gender, age (youths and young adults), and distress groups. Configural invariance models were estimated using multiple-group CFA under robust weighted least square estimator as the baseline model. In the configural model, different factor loadings and thresholds/intercepts were estimated for the items across groups. These parameters were constrained to be equal across the groups in the scalar invariance model (27). Model comparison was conducted *via* the chi-square difference test and the change in model fit indices (40). A decrease of less than 0.01 for ΔCFI and an increase of less than 0.01 for ΔRMSEA and ΔSRMR constituted support for scalar measurement invariance (41). Structural invariance tests examined the equality of factor means across groups. Model identification was achieved by fixing the factor loading at one and comparison of latent means was based on a standardized metric. Third, convergent validity of the PHQ-4 factor was examined *via* its bivariate correlations with the MIL factor, COVID-19 impact, exposure to suicide, self-report distress, and SI. We compared the profiles of participants with and without self-report distress *via* chi-square test and independent *t*-tests using SPSS 26. Effect sizes were indicated by Cohen *d* (42) with cut-off scores of 0.2, 0.5, and 0.8 denoting small, moderate, and large effect sizes, respectively. Statistical significance was set at the 0.05 in the present study.

The fourth step investigated the mediating role of PHQ-4 in the relationship between MIL and SI. Direct and indirect effects of MIL on SI *via* PHQ-4 were evaluated using structural equation modeling (SEM), where the MIL factor, PHQ-4 factor, and SI were specified as latent predictor, latent mediator, and binary outcome, respectively. Gender, age, student status, COVID-19 impact, and exposure to suicide were included as control variables. R-square denoted the proportion of explained variance of the dependent variables. To account for the likely skewed distribution, the indirect effects were estimated using 10,000 bootstrap draws and regarded as statistically significant if the 95% confidence interval (CI) excluded zero. The total natural indirect effect and pure natural direct effect were presented as odds ratios (OR) for the binary SI based on counterfactuals (43). Multigroup SEM was performed to compare the indirect effects of MIL on SI *via* PHQ-4 across the non-distress and distress groups. Logistic regression was performed to examine the factors associated with help-seeking among distressed respondents.

## Results

### Sample profile of young adults

Table 1 reports the sample profiles and item descriptive statistics. Half of the sample (51.7%) were males with a mean age of 26.3 years (*SD* = 3.78). One-tenth (10.1%) of the respondents reported SI and 31.2% of them personally knew someone who attempted suicide before. Half (51.6%) of them encountered distressing issues in the past 4 weeks and 84.7% of them had sought help for the distress. The sample reported average total scores of 2.72 (SD = 2.42) and 4.38 (SD = 1.35) for PHQ-4 and MIL, respectively. SI was negatively correlated with the MIL items (*r* = −0.28 to −0.33, *p* < 0.01) and positively correlated with COVID impact, exposure to suicide, and the PHQ-4 items (*r* = 0.20–0.55, *p* < 0.01).

**TABLE 1:** Descriptive statistics and polychoric correlations of the study variables in the sample.

	Range	M *(SD)*	Skewness	1	2	3	4	5	6	7	8	9	10	11	12
1. Gender (female)	0–1	0.48 (0.50)	/												
2. Age	18–36	26.3 (3.78)	0.60	-0.01											
3. Students	0–1	0.16 (0.37)	/	-0.08	-0.74*										
4. COVID-19 impact	1–5	2.83 (1.21)	0.10	0.05	-0.03	0.08									
5. Exposure to suicide	0–1	0.31 (0.46)	/	0.08	-0.03	0.11	0.07								
6. MIL1	1–7	4.72 (1.50)	−0.30	0.06	0.11*	-0.05	-0.11*	-0.01							
7. MIL2	1–7	3.97 (1.53)	0.05	0.09*	0.12*	-0.05	-0.14*	-0.05	0.67*						
8. MIL3	1–7	4.46 (1.56)	−0.23	0.07	0.09*	-0.03	-0.13*	-0.02	0.64*	0.67*					
9. PHQ1	0–3	0.73 (0.86)	1.15	-0.05	-0.09*	0.09	0.10*	0.09	-0.27*	-0.27*	-0.26*				
10. PHQ2	0–3	0.58 (0.74)	1.33	0.01	-0.11*	0.05	0.14*	0.14*	-0.26*	-0.29*	-0.26*	0.56*			
11. PHQ3	0–3	0.91 (0.78)	0.81	0.11*	-0.04	-0.03	0.13*	0.20*	-0.24*	-0.25*	-0.24*	0.43*	0.63*		
12. PHQ4	0–3	0.50 (0.76)	1.58	0.11*	-0.06	0.12	0.19*	0.12*	-0.22*	-0.25*	-0.22*	0.45*	0.64*	0.73*	
13. Suicidal ideation	0–1	0.10 (0.30)	/	0.02	-0.10	0.14	0.20*	0.37*	-0.33*	-0.31*	-0.28*	0.33*	0.55*	0.39*	0.46*

*N* = 1,472; *p < 0.01; MIL, Meaning in life; PHQ, Patient Health Questionnaire.

### CFA results and measurement invariance

As shown in Table 2, the 2-factor CFA model provided a good approximate fit to the data. The significant chi-square test (*p* < 0.01) could be attributed to the relatively large sample size (*N* > 1,000). Substantial factor loadings were found for the PHQ-4 (λ = 0.65–0.88, *p* < 0.01) and MIL (λ = 0.79–0.85, *p* < 0.01) factors with satisfactory composite reliability (Ω = 0.80–0.86). There was a significant residual correlation (*r* = 0.45, *p* < 0.01) between the two PHQ-4 anxiety items. The configural invariance models provided adequate model fit across gender, age, and distress groups. The chi-square difference tests were significant across gender (χ^2^ = 33.3, *df* = 14, *p* < 0.01) but not across age and distress groups (χ^2^ = 19.4–24.1, *df* = 14, *p* = 0.05–0.15). The scalar invariance models did not show deteriorated fit indices with ΔCFI, ΔRMSEA, and ΔSRMR all falling within ± 0.01. Females showed significantly higher latent means in PHQ-4 and MIL (*d* = 0.15–0.24, *p* < 0.01) than males. Young adults showed significantly lower latent means in PHQ-4 (*d* = 0.18, *p* < 0.01) and higher latent means in MIL (*d* = 0.21, *p* < 0.01) than youths. The distress group showed significantly higher latent means in PHQ-4 (*d* = 1.34, *p* < 0.01) and lower latent means in MIL (*d* = 0.39, *p* < 0.01) than non-distress group.

**TABLE 2:** Fit indices of CFA and SEM of meaning in life and Patient Health Questionnaire-4 in the overall sample and across subgroups.

Model specification	χ ^2^	*df*	RMSEA (90% CI)	stocktickerCFI	TLI	SRMR
Overall 2-factor CFA model	40.1	12	0.040 (0.027–0.054)	0.992	0.987	0.017
**Configural invariance**						
Across gender	41.2	24	0.031 (0.014–0.047)	0.995	0.992	0.017
Across age groups	57.7	24	0.044 (0.029–0.058)	0.991	0.984	0.020
Across distress groups	60.5	24	0.045 (0.031–0.060)	0.987	0.977	0.023
**Scalar invariance**						
Across gender	75.0	38	0.036 (0.024–0.048)	0.990	0.989	0.021
Across age groups	77.2	38	0.037 (0.025–0.049)	0.989	0.988	0.023
Across distress groups	74.8	38	0.036 (0.024–0.048)	0.987	0.985	0.026
SEM in overall sample	110.3	42	0.033 (0.026–0.041)	0.986	0.977	0.043
SEM across distress groups	174.1	100	0.031 (0.023–0.039)	0.980	0.973	0.070

*N* = 1,472; CFA, confirmatory factor analysis; SEM, structural equation model; χ^2^, chi-square; *df*, degree of freedom; RMSEA, root mean square error of approximation; CI, confidence interval; stocktickerCFI, comparative fit index; TLI, Tucker-Lewis index; SRMR, standardized root mean square residuals.

### Convergent validity of PHQ-4 and MIL

The PHQ-4 and MIL factors were negatively and moderately correlated (*r* = −0.41, *p* < 0.01). The PHQ-4 factor was positively correlated with COVID impact (*r* = 0.19, *p* < 0.01), exposure to suicide (*r* = 0.19, *p* < 0.01), and SI (*r* = 0.59, *p* < 0.01). As Table 3 shows, significantly more participants in the distress group were exposed to suicide and had SI (χ^2^ = 40.9–58.3, *p* < 0.01) than the non-distress group. There were significant and small group differences (*d* = 0.21–0.38, *p* < 0.01) in terms of age, COVID-19 impact, and MIL. The distress group showed substantially higher levels (*d* = 0.88, *p* < 0.01) of PHQ-4 than the non-distress group.

**TABLE 3:** Comparison of the profiles across participants with and without self-report distress in the past 4 weeks.

	With distress (*N* = 759)	Without distress (*N* = 713)			
**Categorical variables**	**N (%)**	**N (%)**	**χ ^2^**	***p***	
Gender–Female	368 (48.5)	342 (48.0)	0.04	0.84	
Student status	132 (17.4)	102 (14.3)	2.62	0.11	
Exposure to suicide	294 (38.7)	166 (23.3)	40.9	<0.01*	
Suicidal ideation	121 (15.9)	28 (3.9)	58.3	<0.01*	
**Continuous variables**	**M (SD)**	**M (SD)**	***t***	***p***	***d***
Age	25.9 (3.51)	26.7 (4.01)	-3.95	<0.01*	0.21
COVID-19 impact	3.00 (1.22)	2.65 (1.17)	5.61	<0.01*	0.29
Meaning in life	4.14 (1.37)	4.64 (1.27)	-7.25	<0.01*	0.38
Patient Health Questionnaire-4	3.66 (2.52)	1.72 (1.84)	16.9	<0.01*	0.88

*p < 0.01; χ^2^, chi-square statistic; *t*, *t*-test statistic; *d*, Cohen’s d.

### Results of mediation analysis

As shown in Table 2, the SEM provided an adequate fit to the overall sample. Females showed higher levels of MIL and PHQ-4 (*β* = 0.15–0.17, *p* < 0.05). Age was positively associated with MIL (*β* = 0.04, *p* < 0.01). Student status was not significantly associated (*p* = 0.18–0.78) with the study variables. COVID-19 impact was associated with higher levels of PHQ-4 (*β* = 0.11, *p* < 0.01) and lower levels of MIL (*β* = −0.13, *p* < 0.01). Exposure to suicide was positively associated with PHQ-4 and SI (*β* = 0.30–0.44, *p* < 0.01). MIL showed a negative effect on PHQ-4 (*β* = −0.43, *p* < 0.01) and PHQ-4 had a positive effect on SI (*β* = 0.45, *p* < 0.01). The model explained 21.0 and 41.3% of the variances of PHQ-4 and SI, respectively. Table 4 lists the direct and indirect effects and associated odds ratios of MIL on SI *via* PHQ-4 in the overall SEM. MIL showed significant and negative direct and indirect effects on SI *via* PHQ-4. The PHQ-4 factor accounted for 53.7% of the total effect of MIL on SI.

**TABLE 4:** Direct and indirect effects and associated odds ratios from meaning in life to suicidal ideation *via* PHQ-4 in the overall structural equation model and across distress groups.

Sample	Estimate	95% CI	Odds ratios	95% CI
**Overall sample (*N* = 1,472)**				
Direct effect	−0.169*	−0.275 to −0.062	0.676*	0.475 to 0.875
Indirect effect	−0.196*	−0.254 to −0.144	0.617*	0.489 to 0.726
**Without distress (*N* = 713)**				
Direct effect	−0.256*	−0.440 to −0.084	0.423*	0.147 to 0.774
Indirect effect	−0.063*	−0.124 to −0.008	0.802*	0.583 to 0.975
**With distress (*N* = 759)**				
Direct effect	−0.151*	−0.303 to −0.009	0.697*	0.399 to 0.981
Indirect effect	−0.208*	−0.299 to −0.135	0.590*	0.402 to 0.739

CI, confidence interval; PHQ-4, Patient Health Questionnaire-4; *p < 0.05 with the 95% bootstrapped confidence intervals excluding zero for the direct and indirect effects and excluding one for the odds ratios.

**FIGURE 1:**
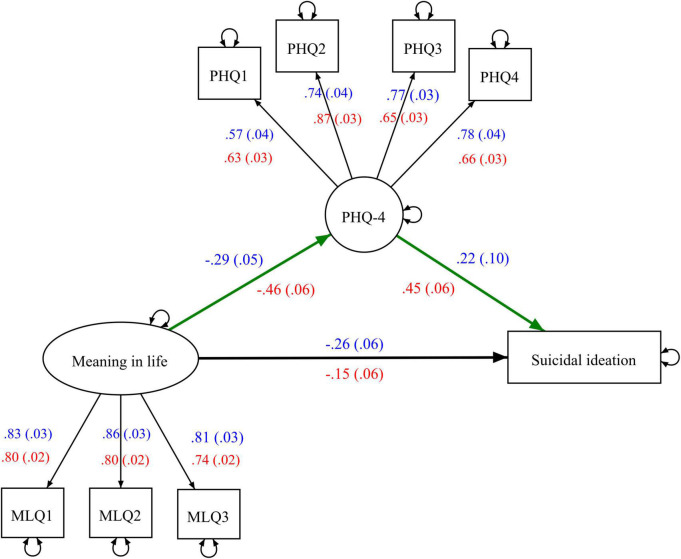
Path coefficients from meaning in life to suicidal ideation (SI) *via* PHQ-4 in structural equation model across non-distress and distress groups. PHQ-4, Patient Health Questionnaire-4; the regression paths and factor loadings in the non-distress and distress groups are shown in blue and red, respectively. Regression paths among the latent variables and SI are bolded and the indirect effects from meaning in life to SI *via* PHQ-4 are highlighted in green.

Figure 1 depicts the factor loadings and regression paths among MIL, PHQ-4, and SI in the multigroup SEM. The negative effect of MIL on PHQ-4 was significantly stronger (Δ = −0.174, *p* = 0.028) in the distress group than the non-distress group. The distress group showed a significantly stronger positive effect of PHQ-4 on SI (Δ = 0.233, *p* = 0.039) than the non-distress group. As Table 4 shows, the distress group showed a significantly stronger indirect effect of MIL on SI *via* PHQ-4 (Δ = −0.146, 95% CI = −0.252 to −0.049) than the non-distress group. The negative direct effect of MIL factor on SI was not statistically significant (Δ = 0.105, 95% CI = −0.121 to 0.335) across the two groups. The PHQ-4 factor accounted for 19.7 and 57.9% of the total effect of MIL on SI in the non-distress and distress groups, respectively.

### Associated factors of help-seeking in the distress group

As shown in Table 5, females were significantly associated with a higher likelihood of help-seeking (90.7%) than males (78.9%). Age, student status, COVID-19 impact, exposure to suicide, PHQ-4, and SI were not significantly associated (*p* = 0.50–0.99) with help-seeking behaviors. Higher MIL was significantly associated with a higher likelihood of help-seeking.

**TABLE 5:** Logistic regression of help-seeking behaviors on associated factors in the distress group.

	B (SE)	Odds ratios (95% CI)
Gender–Female	0.90 (0.22)*	2.46 (1.60–3.80)
Age	−0.01 (0.04)	1.00 (0.93–1.07)
Student status	0.06 (0.31)	1.06 (0.58–1.96)
COVID-19 impact	0.00 (0.08)	1.00 (0.85–1.18)
Exposure to suicide	−0.01 (0.22)	1.00 (0.65–1.54)
Meaning in life	0.38 (0.13)*	1.46 (1.14–1.88)
Patient Health Questionnaire-4	−0.09 (0.13)	0.91 (0.70–1.19)
Suicidal ideation	0.12 (0.30)	1.13 (0.62–2.04)

B, unstandardized regression coefficient; SE, standard error; CI, confidence interval; *p < 0.01.

## Discussion

The present study systematically evaluated the psychometric properties of the PHQ-4 in a large random sample of young adults in Hong Kong. The CFA results demonstrated factorial validity and reliability of the PHQ-4 and MIL factors and supported Hypothesis 1. Consistent with recent studies (24–26, 35), the PHQ-4 and MIL factors showed scalar measurement invariance across gender and age. This lends empirical support to Hypothesis 2. In addition, both factors demonstrated scalar measurement invariance across distress groups in the Chinese context. The scalar measurement invariance enables unbiased and meaningful comparisons of the latent levels between young adults with and without distress. The PHQ-4 factor showed significant correlations with MIL, SI, COVID impact, and exposure to suicide in the expected directions. The large differences in the latent and observed PHQ-4 scores across the distress groups support Hypothesis 3 in terms of adequate convergent validity. The SEM results provided evidence for Hypothesis 4 and the PHQ-4 mediated around half of the total effect from MIL to SI in the overall sample.

Consistent with previous findings (13, 18, 19), our findings provided empirical support on the protective role of presence of MIL against SI. The findings not only demonstrate the potential clinical relevance of using PHQ-4 as a brief and direct screening tool of SI, but also inform clinical practice on refinement of SB risk assessment *via* a meaning-oriented approach (44). Multigroup SEM revealed significantly greater negative indirect effects of MIL on SI *via* PHQ-4 in the distress group than the non-distress group. This finding implies a stronger mediating role for PHQ-4 in the distress group, in which over half of the prevention effect of SI could be attributed to the capacity of MIL to lower the psychological distress. In comparison, the PHQ-4 only accounted for one-fifth of the effect of MIL on SI in the non-distress group. Compared to the PHQ-4, the three MIL questions inquired the general well-being of the respondents and MIL could be more relevant to the general population with comparably better mental health. More than half of the young adults encountered distressing issues in the past 4 weeks and the majority of them had sought help for their distress. One should note that the prevalence of suicide ideation in the distress group was four times as high as in the non-distress group. The increased exposure to suicide, greater COVID-19 impact, and lower MIL point to greater service needs for these distressed individuals.

The present findings have important implications that could influence and clinically guide the practical implications. COVID-19 impact was found to be indirectly related to mental health distress *via* MIL in a recent study (14). Our findings contributed to the literature by demonstrating that elevated levels of psychological distress further led to SI. In a study among 1,477 psychiatric inpatients in Switzerland (45), distinct pandemic-related fear patterns could manifest during lockdown periods under the COVID-19 pandemic in the form of loneliness and isolation, loss of work, feelings of powerlessness. COVID-19 related fears could act as an underlying source of psychological distress and lead to mental health symptoms. Another large-scale study among 8,177 university students in Italy (46) found social isolation due to the pandemic to contribute to psychological distress in young people. The mental health symptoms could be linked to interpersonal factors in the young adults related to suicidality.

In contrast to previous findings (47), help-seeking behaviors were not significantly associated with SI and PHQ-4 in the distress group. Rather than shortage of mental health professionals, lack of perceived needs for treatment could be the primary reason why persons with psychological distress chose not to seek help (48). Suicide stigma could act as barriers to seek help among the distressed individuals (49). Nguyen, Le (47) found informal and formal help-seeking to be negatively and positively associated with SI, respectively. It would be of practical importance to distinguish the patterns and motives of different help-seeking behaviors (37). Given the potential benefits of online help-seeking behaviors to bridge barriers to traditional help-seeking (48), tailored preventive strategies are recommended to evaluate the feasibility of providing early intervention for the vulnerable youths *via* online emotional support programs. Magson, Freeman (50) examined the protective factors for temporal changes in mental health of the adolescents during the COVID-19 pandemic. Future longitudinal studies could explore the moderating role of MIL (51–53) on other protective factors such as resilience (54) in the progression of SI (55) and development of SA (56).

The present study has a number of strengths. First, young people are often prone to question the value system and MIL could contribute substantially to their well-being (57). Despite the potential importance and clinical implications, the relationship between MIL and SI remains undervalued in the current literature. The present study addressed a topical research question by focusing on the population of young adults as one of the vulnerable groups with regard to SI and SB under the COVID-19 pandemic. Second, we recruited a large sample of young adults *via* random sampling in Hong Kong. The random sampling design and moderately high response rate lent support to the representativeness of the sample. Third, SEM is a latent variable modeling technique that accounts for the measurement errors of the constructs and offers more precise estimates of the regression coefficients. Since ignored measurement errors in the mediator could distort the direct and indirect effects, the use of factor analysis model for multiple indicators of the MIL and PHQ-4 as latent variables strengthened the credibility of the results (43).

Several limitations should be noted about the present study. First, cross-sectional study design did not permit examination of the test-retest reliability and temporal stability of the factor structure. Longitudinal studies are recommended to explore the longitudinal measurement invariance of the PHQ-4 and track the temporal change in PHQ-4 over time. Second, the sample respondents only reported SI but not SA. A large-scale cohort study found that exposure to self-harm in others and diagnosis of psychiatric disorder differentiated the suicide attempters from the suicide ideators (58). Future longitudinal studies are needed to differentiate between suicide ideators and suicide attempters and elucidate risk factors that contribute to progression from SI to SA. Third, the present study focused only on young adults and our results may not be generalized to other age groups. Future studies are needed to elucidate the potential protective role of MIL as a resiliency factor to SI among older adults as in Beach et al. (59) and Heisel et al. (60).

Fourth, the present sample only completed the PHQ-4 but not the PHQ-9. We could not test the comparability of the PHQ-4 and PHQ-9 scores. The phone sampling design did not allow us to assess the criterion validity of the PHQ-4. Further studies are needed to compare the diagnostic ability of the PHQ-4 on psychiatric morbidity with other established scales. Fifth, the present SEM only controlled for the effects of COVID-19 impact, exposure to suicide, and self-report distress. Future studies should include other risk factors of suicide such as bullying victimization and self-harm. Sixth, the impact of COVID-19 on the respondents was only assessed *via* a single item, which was subject to concerns such as unreliability and low content validity. Validated scales such as the Tokyo Metropolitan Distress Scale for Pandemic (61) and Mental Impact and Distress Scale: COVID-19 (62) should be used as reliable measurement of pandemic-specific impact.

## Conclusion

The present study contributes to a better understanding of the psychometric properties of the PHQ-4 in a large sample of young adults in Hong Kong. The results provide empirical support for adequate factorial validity, reliability, convergent validity, and scalar measurement invariance across gender, age, and distress groups. The PHQ-4 factor demonstrated a substantial mediating role in the relationship between MIL and SI in the overall sample and the distress group. The use of PHQ-4 is recommended as a valid and reliable measure of general psychological distress in future studies in the Chinese context.

## Data availability statement

The original contributions presented in this study are included in the article/Supplementary material, further inquiries can be directed to the corresponding authors.

## Ethics statement

The studies involving human participants were reviewed and approved by Human Research Ethics Committee of the University of Hong Kong (HREC Number = EA1709039). The patients/participants provided their written informed consent to participate in this study.

## Author contributions

TF: conceptualization, data curation, formal analysis, literature review, methodology, validation, visualization, and writing—original draft. RH: conceptualization, software, resources, and writing—review and editing. PY: conceptualization, investigation, project administration, methodology, writing—review and editing, supervision, and funding acquisition. All authors contributed to the article and approved the submitted version.
